# Huge arachnoidal cyst with recent onset of seizure in an adult

**DOI:** 10.11604/pamj.2023.45.84.38644

**Published:** 2023-06-14

**Authors:** Mohamed El Biadi, Salah Bellasri

**Affiliations:** 1 Department of Radiology, Avicenne Military Hospital of Marrakech, Faculty of Medicine and Pharmacy of Marrakech, Cadi Ayyad University of Marrakech, Marrakech, Morocco

**Keywords:** Huge arachnoidal cyst, onset of seizure, adult, brain imaging

## Image in medicine

A 68-year-old right-handed man, having consulted for the occurrence of a first epileptic seizure. Brain computerized tomography (CT) scan and magnetic resonance imaging (MRI) showed a giant congenital supratentorial arachnoidal cyst occupying the anterior two-thirds of the right hemisphere, measuring 14.09x6.07 (APxT) cm and 11.67 cm high, compressing the right cerebral hemisphere and the right cerebral peduncle with a midline shift. Arachnoidal cysts are congenital malformations containing cerebrospinal fluid. They may be asymptomatic and discovered incidentally on imaging. When they are large, as in this patient, they can cause a mass effect.

**Figure 1:**
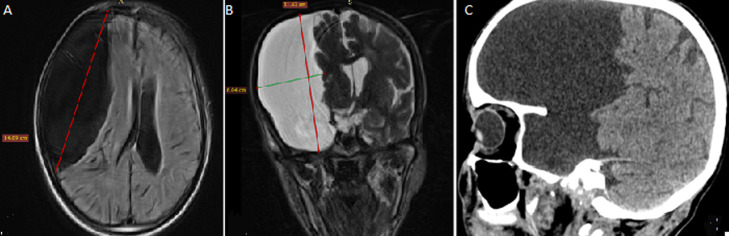
magnetic resonance imaging of the brain (axial T1-weighted; A) and coronal T2-weighted; B) cerebral computerizing tomography scan in spontaneous contrast, sagittal section; C) huge compressive arachnoidal cyst of the non-dominant hemisphere

